# The cost-effectiveness of government actions to reduce sodium intake through salt substitutes in Vietnam

**DOI:** 10.1186/s13690-021-00540-4

**Published:** 2021-03-11

**Authors:** Colman Taylor, Annet C. Hoek, Irene Deltetto, Adrian Peacock, Do Thi Phuong Ha, Michael Sieburg, Dolly Hoang, Kathy Trieu, Laura K. Cobb, Stephen Jan, Jacqui Webster

**Affiliations:** 1 The George Institute for Global Health, Australia University of NSW, PO Box M201, Missenden Rd, Camperdown, NSW 2050 Australia; 2 Health Technology Analysts Pty Ltd, Surry Hills, Australia; 3 National Institute of Nutrition, Hanoi, Viet Nam; 4 YCP Solidiance Company Ltd, Hanoi, Viet Nam; 5 Resolve to Save Lives, An Initiative of Vital Strategies, New York, NY USA

**Keywords:** Diet, Sodium, CHD, Stroke, Cost-effectiveness, Health economics

## Abstract

**Background:**

Dietary sodium reduction is recommended to reduce the burden of cardiovascular disease. In Vietnam food products including salt, fish sauce and bot canh contribute to ~ 70% of dietary sodium intake. Reduced sodium versions of these products can be produced by replacing some of the sodium chloride with potassium chloride. We aimed to assess the cost-effectiveness of three alternative approaches to introducing reduced sodium products onto the market with a view to lowering population sodium intake in Vietnam.

**Methods:**

The three salt substitution strategies included voluntary, subsidised and regulatory approaches targeting salt, fish sauce and bot canh products. Costs were modelled using the WHO-CHOICE methodology. A Markov cohort model was developed to evaluate the cost-effectiveness of each strategy versus no intervention from the government perspective. The model linked each intervention strategy to assumed changes in levels of sodium intake and then to systolic blood pressure. Changes in SBP were linked to a probability of ischaemic heart disease or stroke. The model followed people over their lifetime to assess average costs and quality adjusted life years (QALYs) gained for each strategy.

**Results:**

The voluntary salt substitution strategy was assumed to require no investment by government. Following ramp up (years 6+), the average annual costs for the subsidised and regulatory strategies were 21,808,968,902 ₫ (US$ 977,354) and 12,949,953,247 ₫ (US$ 580,410) respectively. Relative to no intervention, all three salt substitution strategies were found to be cost-effective. Cost savings were driven by reductions in strokes (32,595; 768,384; 2,366,480) and ischaemic heart disease (IHD) events (22,830; 537,157; 1,648,590) for the voluntary, subsidised & regulatory strategies, respectively. The voluntary strategy was least cost-effective (− 3445 ₫ US$ -0.15; 0.009 QALYs gained) followed by the subsidised strategy (− 43,189 ₫ US$ -1.86; 0.022 QALYs gained) and the regulatory strategy delivered the highest cost savings and health gains (− 243,530 ₫ US$ -10.49; 0.074 QALYs gained).

**Conclusion:**

This research shows that all three modelled salt substitution strategies would be good value for money relative to no intervention in Vietnam. The subsidised alternative would require the highest level of government investment; however the implementation costs will be exceeded by healthcare savings assuming a reasonable time horizon is considered.

**Supplementary Information:**

The online version contains supplementary material available at 10.1186/s13690-021-00540-4.

## Background

Cardiovascular disease (CVD) is one of the leading causes of non-communicable disease mortality and morbidity globally [1]. The most common causes of CVD morbidity and mortality are ischaemic heart disease (IHD) and stroke, accounting for 85% of all CVD deaths in 2016 [2, 3]. Due to higher exposure to modifiable risk factors and poor access to effective health care interventions, the impact of CVD is magnified in lower income countries [1].

The direct causal relationship between dietary salt (sodium chloride) intake and blood pressure is now well established [4]. It has long been recognised that hypertension (high blood pressure) is one of the major risk factors for stroke and IHD [5, 6]. Reduction of dietary salt intake is considered an effective measure to reduce blood pressure, with the World Health Organisation (WHO) recommending the consumption of less than 5 g (g) of salt per day [7]. The WHO has urged its member states to take action at a population level to reduce dietary salt intake [8]. The reduction of excess dietary salt is widely recognised as one of the most cost-effective means for lowering blood pressure and preventing non-communicable disease across the world [9–18].

With economic development and an aging population, Vietnam has undergone a transition from managing communicable diseases to now confronting a growing population suffering from non-communicable disease [19, 20]. The WHO estimates that non-communicable diseases account for 73% of total deaths in Vietnam, of which approximately 43% are attributed to cardiovascular diseases including IHD and stroke [21]. Hypertension has been one of the primary contributors to the overall burden of disease in Vietnam during this shift. In 1960, the rate of adult hypertension in Northern Vietnam was 1% [22]. The prevalence of hypertension in Vietnam as of 2014 is estimated to be 22% among adults [21]. This has significant implications for the Vietnam Government as the main funder of healthcare.

Recent research has estimated the average salt intake in Vietnam to be 9.4 g/day [23], nearly double the 5 g/day recommended by the WHO [8]. Approximately 70% of salt consumption currently comes from salt, fish sauce and bot canh [24]. One method of reducing sodium in these food products is the use of a salt substitute, such as potassium chloride, which has been shown to be effective at lowering blood pressure [25]. Various options exist to implement salt substitution at a population level, including leaving manufacturers to reformulate products (in a voluntary capacity) or involving Government (through subsidies or regulation). While studies conducted both globally and locally in Vietnam have found salt reduction policies and campaigns to be very cost-effective [4, 13, 17, 26, 27], the cost-effectiveness of a salt substitution intervention using potassium-enriched low sodium food products, from the perspective of the Vietnam Government, remains unknown.

We aimed to assess the cost-effectiveness of reformulating three target products that make up the majority of salt intake in Vietnam with potassium chloride and therefore lowering sodium intake in Vietnam at a population level. In doing so, we investigate the cost of three potential salt substitution strategies and a range of effectiveness estimates, to estimate the cost-effectiveness of salt substitution at a population level in Vietnam.

## Methodology

**Fig. 1:**
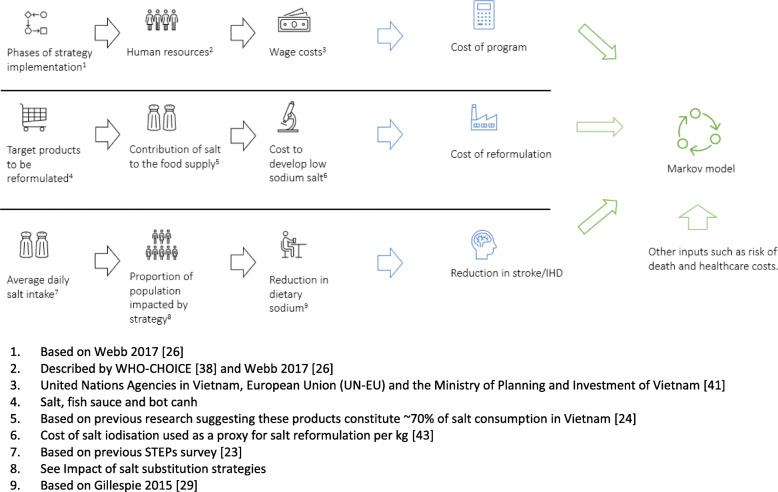
Schemata of development of key assumptions to be incorporated into the cost-effectiveness model

Three salt substitution strategies using potassium chloride were investigated to reduce sodium in three target products in Vietnam (salt, fish sauce and bot canh [a popular seasoning in Vietnam]). All strategies were detailed and costed both in terms of program costs and salt reformulation costs. Population impact of the strategies was also estimated based on the proportion of products reformulated and the adoption of the low salt products by consumers. Inputs including program costs, reformulation costs, program impact and other variables were then modelled to understand the cost-effectiveness of the salt substitution strategies (Fig. 1).

### Definition of salt substitution strategies

The three salt substitution strategies included voluntary, subsidised and regulatory approaches as proxies for low, medium and high impact interventions respectively (see Tables and figures Table 1). These would lead to the consumers replacing a proportion of the target product consumption with potassium-enriched, low sodium options.

**Table 1:** Details of salt substitution strategies including estimated uptake, cost, resources and coverage

Parameter	Voluntary	Subsidised	Regulatory
Description	Companies that make target products* voluntarily introduce new products low sodium products with potassium chloride.	Government subsidisation of potassium chloride to equal cost of salt – allowing manufacturers to use potassium chloride in target products* as a salt substitute.	Legislation requiring all target products* to have a portion of their sodium content replaced by potassium chloride.
Industry uptake	Assumed to be low	Assumed to be moderate	Assumed to be high
Cost	Extra cost of low sodium products is passed onto consumers	Borne by Government (for subsidy). Manufacturers are assumed to produce and sell low salt products at a similar price to regular products and therefore no extra cost is passed onto consumers	Borne by Government (for legislation) and manufacturers (for production). Low salt products are assumed to be sold at a similar price
Proportion of target products* reformulated with potassium chloride	30%	50%	100%
Proportion of people choosing reformulated target products*	5%	44%	100%
Media and communications campaign	Not included	Included	Not included

Briefly, the voluntary scenario assumes companies will voluntarily provide new reformulated low sodium products. This requires no government support (low cost) but is assumed to have a lower uptake by consumers and therefore lower impact on population level sodium consumption (see Impact of salt substitution strategies). Both the subsidised and regulatory scenarios require government intervention to either incentivise or mandate food companies to reformulate existing target products with potassium chloride. In the subsidised scenario, the Vietnam Government is assumed to fund reformulation of a proportion of the target products, whereas in the regulatory scenario, the Vietnam Government will mandate that all target products are reformulated with potassium chloride.

As voluntary strategy assumes that initiatives are left to the market and food industry with no involvement or coordination from Government, no coordinated mass media campaign was included. In contrast, the subsidised strategy included a communications and media campaign to drive uptake, as stakeholder research with manufacturers indicated this would be essential to justify R&D costs [28]. Finally, the regulatory strategy included no media campaign as compliance was assured through regulation. This is consistent with a similar scenario modelled for China [4].

### Calculation of impact of salt substitution strategies

The decrease in sodium chloride content in food products due to substitution with potassium chloride for each modelled strategy is based on the coverage, efficacy and impact formula described by Gillespie et al. [29]. The method takes into consideration the proportion of daily sodium intake from the low sodium target products, the effect of reformulation, the proportion of targeted products to be reformulated and the expected uptake of the low sodium products. This provides an estimated daily reduction in sodium consumption which can be applied to the average daily sodium intake (Table 2).

**Table 2:** Details of assumptions for each salt substitute strategy to calculate impact on average dietary salt intake, Vietnam

Parameter	Voluntary	Subsidised	Regulatory	Source
Average daily salt intake (A)	9.4 g/day	9.4 g/day	9.4 g/day	[23]
Proportion of salt from target products (B)	70%	70%	70%	[24]
Sodium reduction in target products due to reformulation	60%	60%	60%	[30]
Proportion of target products reformulated with potassium chloride (C)	30%	50%	100%	Assumptions
Proportion of people choosing reformulated target products (D)	5%	44%	100%	[31]
Dietary salt reduction due to reformulation (E)	0.058 g/day	0.865 g/day	3.95 g/day	A x B X C X D Gillespie 2015 [29]
Full effect of strategy on average daily salt intake	9.34 g/day	8.53 g/day	5.45 g/day	A - E

An additional 5% reduction from baseline was added to the subsidised intervention to reflect the additional benefit of a communications intervention to reduce salt intake in Vietnam; target products include salt, fish sauce, bot canh

Recognising the uncertainty of these assumptions and their impact on the model result, a threshold analysis was conducted (see Sensitivity and Threshold Analysis). The model does not consider any beneficial or harmful effects stemming from potassium chloride.

All strategies started from the average salt intake for the Vietnamese population, which was estimated to be 9.40 g/day [23]. The salt reduction in target products due to reformulation was consistent throughout the strategies (60% based on previous research [30]).

For the voluntary strategy, it was assumed that manufacturers would choose to reformulate 30% of the target products (salt, fish sauce and bot canh). However due to reformulation costs being passed onto consumers, it was assumed uptake of these products would be low (5%). To ascertain the impact of this strategy, the daily salt intake (9.4 g) was multiplied by the following variables per Gillespie et al. [29]: the proportion of dietary salt from the target products (70%); the salt reduction in target products due to reformulation (60%); the proportion of products reformulated (30%); the product uptake (5%). This resulted in a modest total dietary salt reduction of 0.06 g/day (Table 2).

In contrast, the regulatory strategy was assumed to have maximum uptake as legislation would guarantee all target products are reformulated with potassium chloride, which would lead to 100% of people having to use the reformulated products. The latter assumption takes into account the high adoption rate (> 90%) seen in the Vietnam National Iodisation Program [32]. The impact of the regulatory strategy on dietary salt intake was calculated by multiplying the baseline daily salt intake (9.4 g/day) by the following variables: the proportion of dietary salt from the target products (70%); the salt reduction in target products due to reformulation (60%); the proportion of products reformulated (100%); the product uptake (100%). This resulted in a total dietary salt reduction of 3.95 g/day (Table 2).

For the subsidised intervention, it was assumed 50% of the target products would be reformulated with potassium chloride. It was assumed a government subsidy would be used to supply potassium chloride at the same price or cheaper than sodium chloride. This would lead to reformulated products being sold at the same price or cheaper than regular products. Product uptake of the reformulated products was based on a Vietnamese population survey that found approximately 44% of respondents would limit adding salt or sauces when cooking when given the option [31]. Furthermore, the media and health promotion campaign accompanying the subsidised programme is expected to have an additive effect on the intake of high-sodium products and is reflected as an extra 5% reduction from baseline [13].

The impact of the subsidised strategy on dietary salt intake was calculated by multiplying the baseline daily salt intake (9.4 g/day) by the following variables: the proportion of dietary salt from the target products (70%); the salt reduction in target products due to reformulation (60%); the proportion of products reformulated (50%); the product uptake (44%). This resulted in a total dietary salt reduction of 0.87 g/day (Table 2).

### Assumptions for strategy implementation

To reflect the real-world planning and management of a population health intervention, each salt substitution strategy included progressive phases of implementation, modelled on Webb 2017 [26]. Specifically, the first phase includes 2 years of project management, training and meetings, advocacy and law enforcement prior to deploying the programme. These years are dedicated to the planning and development of the intervention and therefore no health effects are assumed to take place. Following this stage, the voluntary and subsidised strategy included a partial implementation phase, resulting in 50% of the total salt reduction effect of each respective strategy, to account for progressive uptake. The regulatory strategy assumes that from year three the total effect of the programme would be realised, as once legislation is implemented the programme should be 100% effective (leveraging experience from the Vietnam National Iodisation Program [32]). From years six onwards, all programmes are assumed to be at full implementation.

### Cost-effectiveness model structure and perspective

A Markov cohort model was developed in TreeAge Pro based on approaches adopted in previous publications [13, 17, 26]. The model included four health states, namely healthy, post-stroke, post-IHD and death. Annual transitions captured the incidence of stroke and IHD events, mortality due to IHD or stoke events and natural mortality.

The model starts with people aged 30 years being assigned to live normally in the current environment (no intervention), or alternately, live with one of three salt substitution strategies (voluntary, subsidised or regulatory). A base case age of 30 years was selected as being representative of the median age of the Vietnam population and as it had been used previously to assess cost-effectiveness of salt reduction in Vietnam [13]. At the beginning of the model, the average SBP is calculated according to the Vietnamese population characteristics [13, 33]. The model links sodium intake to SBP based on the linear regression model published by Law et al. 1991 [34]. Secondly, SBP is linked to the probability of IHD or stroke based on Cobiac et al. 2012 [17].

Patients that experience a stroke or IHD event either die as a result of the event or progress to a “post event” health state. In the post-stroke health state, patients have an increased mortality risk compared to the healthy cohort for the lifetime of the model, whereas in the post-IHD cohort there is an increased mortality risk for 3 years post event, after which mortality reverts to the natural mortality risk of that age (see input parameters). As the model has a yearly cycle length, the acute phase (initial, short-term event) for IHD and stroke is captured in a state transition, and the chronic phase (post event, long term) of the disease is captured in the respective health states. Patients cannot transition from the post-stroke or post-IHD state to the healthy state, and conservatively it was assumed patients cannot experience more than one stroke or IHD event.

The time horizon of the economic model spans the lifetime of the Vietnamese population (capped at 100 years of age). A lifetime horizon (which follows a cohort from age 30 until death) was used to fully capture the benefits and costs associated with the salt substitution strategy. The model uses a discount rate of 3% for both benefits and costs; rates of 0 and 5% were used in sensitivity analysis. Given the aims of the study, this population wide model evaluates each scenario from a Vietnam Government perspective and excludes costs borne by industry or individuals.

### Cost-effectiveness model input parameters

A summary of key model inputs is presented in Table 3.

**Table 3:** List of inputs and sources used in cost-effectiveness model, Vietnam

Variable name	Input	Source/Description
*Clinical events and epidemiology*		
Blood pressure	See Supplementary Table 1 Supplementary Material	Calculated based on Ha 2011 baseline blood pressure. Reduction in SBP for each intervention was calculated from the reduction of sodium intake with a linear regression using the Law 1991 SBP with no sodium in the diet as reference [13]
Stroke incidence	See Supplementary Table 2 Supplementary Material	Ha 2011 [13]
Relative risk of stroke with change in SBP	See Supplementary Table 3 Supplementary Material	Cobiac 2012 and intervention specific change in blood pressure from baseline. Each 1% decrease in SBP equals a 6.3% risk reduction for stroke [17]
IHD incidence	See Supplementary Table 2 Supplementary Material	Ha 2011 [13]
Relative risk of IHD with change in SBP	See Supplementary Table 4 Supplementary Material	Cobiac 2012 and intervention specific change in blood pressure from baseline. Each 1% decrease in SBP equals a 3.4% risk reduction for IHD [17]
Mortality	Vietnam life tables	World Health Organisation and Global Health Observatory; age and gender specific [35]
Mortality following stroke event	37%	Tirschwell 2012 [36]
Long term stroke mortality risk	Year 1: 3.33Year 2: 2.85Year 3: 3.44Year 4: 2.84Year 5+: 1.56	Kiyohara 2003 [37]. Relative risk of patients with history of stroke compared to healthy controls. Model assumes patients have elevated risk of mortality (1.56x higher) compared to “healthy” population
Mortality of IHD event	Age specific mortality risk	Southeast Asian NCD impact module dataset through the WHO-CHOICE OneHealth tool
Long term IHD mortality	Year 1: 18.7%Year 2: 25.0%Year 3: 39.2%Year 4+: Revert back to regular population mortality	Tang 2007 [38]. Model assumes that after Year 3 patients have same mortality risk as rest of “healthy” population
*Resource use and programme costs reported in ₫ (USD)*		
Cost of lowering sodium content by potassium-enriched salt substitutes per capita	1791 ₫ (US$ 0.08)	Calculated as the cost of a sodium reduction Government subsidy included in the subsidised scenario. Based on:- 534,798 t of salt produced each year [39]- 70% of salt is in cooking salt, fish sauce and bot canh of which 50% of sodium varieties [24]- US$0.04 to develop 1 kg of low sodium salt [40]
Personnel Costs for policy implementation and management	Project coordinator, manager, chief accountant, technical specialist etc.: 511,526,874 ₫ (US$ 22,039) per yearProject administrative assistant/secretary, accountant, interpreter, translator: 295,489,873 ₫ (US$ 12,730.88) per yearClerk, Driver, Auxiliary Staff, Messenger, Cleaner: 155,828,979 ₫ (US$ 6714) per yearPer diem daily subsistence allowance: 4,015,413 ₫ (US$ 173.00)	UN-EU 2015 human resource costs inflated to 2019 US$ and converted to ₫ [41].Per diem costs from the International Civil Service Commission [42]
Human resource requirements for policy implementation and management	Webb 2017 eTable2	Webb 2017 [26]
*Healthcare costs*		
Percent of healthcare costs paid by the Government	54%	Local expert opinion; WHO 2018 [43]
Cost of stroke event to Government	13,325,677 ₫ (US$ 574.12)	Khiaocharoen 2012 (one off event cost) [44]
Long term cost of stroke to Government	0	Nguyen 2016 identifies stroke patients are cared for at home by family members [45]
Cost of IHD event to Government	17,297,679 ₫ (US$ 745.25)	Nguyen 2016 (one off event cost) [45]
Long term cost of IHD to Government	368,835 ₫ (US$ 15.89)	Nguyen 2016 recurring yearly cost for the lifetime of the patient [45]
*Quality of life*		
Healthy utility (SBP < 130)	Male: 0.734Female: 0.712	Nguyen 2015 [46]
Stage 1 hypertension utility (SBP > 130 and < 140)	Male 0.726Female: 0.705	Nguyen 2015 [46]
Stroke event disutility	− 0.312	GBD 2010 [47]
Long term post-stroke utility	Year 1: 0.66Year 2+: 0.68	Luengo-Fernandez 2013 [48]
IHD event disutility	− 0.186	GBD 2010 [47]
Long term post-IHD utility	OR = − 0.004	Nguyen 2015 odds ratio of patients who had a history of experiencing a cerebrovascular event compared to those without event. Applied to life of patient [46]

Abbreviations: *IHD* ischaemic heart disease, *GBD* Global Burden of Disease study, *SBP* systolic blood pressure

#### Clinical events and epidemiology

Normative data for blood pressure and the incidence of stroke and IHD were sourced from Ha 2011 [13] (see Supplementary Table 1 and 2). This paper provided the most applicable data characterising the relationship between blood pressure and incidence of IHD/stroke for the Vietnamese population. The impact of sodium intake on blood pressure was estimated based on published research by Law 1991 [34], which aligns with previous economic evaluations. The relationship between blood pressure and stroke/IHD was calculated based on the percentage relative risk reduction of stroke (6.3% per 1% SBP reduction) and IHD (3.4% per 1% SBP reduction) as published by Cobiac 2012 [17]. The resulting relative risk reduction for each sodium reduction program is provided in the Supplementary Material (see Supplementary Table 3 and 4).

At any point in the model, patients are assumed to be at risk of death. This represents the probability that a person of a specific age will die before their next birthday. All-cause mortality was sourced from the Global Health Observatory data repository Vietnam life tables, stratified by age and sex [35].

The incidence of mortality following stroke was sourced from Trishwell 2012 [36] and Kiyohara 2003 [37]. The former provided short-term mortality following the event (estimated to be 37%) the latter provided the long term risk of mortality relative to the general population, stratified by year. The risk of mortality following IHD was sourced from the Southeast Asian NCD impact module dataset through the WHO-CHOICE OneHealth tool [49] as well as Tang 2007 [38]. The former provided the age specific mortality risk following IHD and the latter provided long term mortality risk, stratified by year. It was assumed after Year 3 patients have same mortality risk as the rest of the “healthy” population.

#### Cost inputs

All costs were estimated from a Government perspective in 2019 Vietnamese Dong (VND) at an exchange rate of US$1 = 23,210 ₫ [50]. The purchasing power parity (PPP) of 2019 VND from 2015 US$ was calculated to be 7792 using the CCEMG – EPPI-Centre Cost Converter [50].

Given the Government’s minimum involvement in the voluntary strategy, it was assumed no programme implementation costs would be accrued. It is recognised that these costs would be borne by consumers, however this was excluded from the analysis.

Programme costs for the regulatory and subsidised strategies include resources required in the planning, development, and implementation of a population-based health intervention as described by the WHO-CHOICE methodology [49]. This includes the estimated unit price of human resources, training, meetings, supplies, equipment and mass media campaigns (for subsidised strategy only). The resource needs for the regulatory and subsidised interventions were assessed at both a national and provincial level, to reflect regional nuances in cultural and dietary behaviours between provinces. These needs were based on Webb 2017 [26]. Personnel payment norms were based on unified cost norms as issued by United Nations Agencies in Vietnam, European Union (UN-EU) and the Ministry of Planning and Investment of Vietnam [41], inflated to 2019 VND. A per diem daily subsistence allowance of US$173 for attendees of meetings and training was applied, accounting for a travel allowance [42]. The cost of media and communications for the subsidised scenario was incorporated at both a national and provincial level, based on research by Ha 2011 [13].

In addition to programme management costs, the cost of a government subsidy for potassium chloride was included in the subsidised strategy. According to the Vietnamese Ministry of Agriculture and Rural Development, Vietnam produces approximately 534,798 t of salt for human consumption each year [40]. Previous research indicated approximately 70% of salt intake comes from bot canh, fish sauce and salt added when cooking [24]. The literature pertaining to the cost of substituting sodium chloride with potassium chloride is scarce, and as a result, the cost of salt iodisation ($0.04USD per kg) was used as a proxy to estimate the cost of salt reformulation per kilogram [40]. The resulting cost estimate was 1791₫ per capita, reflecting a proxy for the cost of providing potassium chloride for manufacturers at a similar price to regular salt. As the process of salt substitution with potassium chloride is more complex than salt iodisation this assumption is assessed in a sensitivity analysis.

Healthcare costs were derived from Vietnamese specific publications [45, 51] with the exception of the cost of acute stroke event which was not available from Vietnamese sources and therefore obtained from a Thai study [44]. To align with incidence data, the cost of IHD was calculated for the acute event, and for the long term recurring cost of chronic treatment post initial health event [45]. There was no long term cost of stroke applied in the model as rehabilitation and long term care is commonly done by family members at home [45].

According to the WHO, the Vietnamese Government pays for approximately 54% of total healthcare expenditure which was verified by a local source [43]. To reflect a Government perspective, all healthcare costs were calculated accordingly.

#### Quality of life

Quality of life values for healthy, post-stroke and post-IHD health states were applied in the model, and disutility values for stroke and IHD events were applied at health state transitions.

Quality of life values for the Vietnamese population were gender specific for the whole cohort according to blood pressure status, including patients having “ideal” SBP (< 130 mmHg) or Stage 1 SBP (> 130 mmHg) [46].

The long term stroke utility values were sourced from a UK disease-specific population study of the quality of life of patients post-stroke [48]. The quality of life of patients in the post-IHD health state was calculated using a Vietnamese population specific odds ratio comparing the utility of patients with and without history of previous cerebrovascular event [46], which has previously been used as a proxy for the long term quality of life in patients with stable cardiovascular disease [45].

Disutility values for acute stroke and IHD events were sourced from the Global Burden of Disease study [47]. Disutilities represent the decrement in quality of life due to symptoms or events associated with stroke and IHD events. In the model, disutilities are applied once at the time of the event, as well as a recurring disutility that signifies the long term quality of life lost postacute event. When someone experiences a stroke, their immediate quality of life markedly lowers compared to someone who is healthy, thus they receive a disutility of − 0.312 [47]. Similarly for IHD, a person is assigned a one off quality of life decrement of − 0.186 when experiencing the acute IHD event [47]. Those who suffer a stroke or IHD event will often experience long term impacts on their quality of life, referred to in the model as the post-IHD and post-stroke health states.

#### Sensitivity and threshold analyses

A number of one-way sensitivity analyses were run to identify key model drivers and assess any uncertainties. The parameters tested and each upper and lower variable are listed in Table 4.

**Table 4:** List of parameter base case values and ranges used in the sensitivity analyses in the cost-effectiveness model

Parameter	Lower	Base case	Upper
% Products sodium reduced	−10%		10%
Discount rate costs	0%	3%	5%
Discount rate QALYs	0%	3%	5%
Incidence of stroke	−10%		10%
Stroke RR per 1% SBP Δ	4.0%	6.3%	8.0%
Incidence of IHD	−10%		10%
IHD RR per 1% SBP Δ	1.0%	3.4%	5.0%
Event cost Govt: Stroke	0 ₫	12,765,544 ₫	23,596,200 ₫
Event cost Govt: IHD	0 ₫	17,297,680 ₫	31,973,530 ₫
Long term Govt cost: Stroke	0 ₫	0 ₫	25,796,954 ₫
Long term Govt cost: IHD	0 ₫	368,835 ₫	681,766 ₫
Salt substitution cost/kg	US$ 0.02	US$ 0.04	US$ 0.08
Cost of healthcare to Govt	0%	54%	100%
Cost of project implementation	−20%		20%
Disutility stroke event	−0.25	−0.31	−0.40
Disutility IHD event	−0.10	− 0.19	− 0.28
Utility long term stroke	−10%		10%
Utility long term IHD	−10%		10%
Mortality of stroke	−10%	0.37	10%
Mortality of IHD	−10%		10%

Abbreviations: *IHD* ischaemic heart disease, *QALYs* quality adjusted life years, *SBP* systolic blood pressure

In addition, the uncertainty surrounding the impact of the individual salt substitution strategies was recognised and tested in a threshold analysis. Specifically, the average salt reduction needed for average costs to equal cost savings was tested at varying time horizons.

#### Validation

In order to validate the model results, a comparison was undertaken between the model assumptions and results in relation to previous publications including Ha 2011 [13], Webb 2017 [26] and Cobiac 2010 [18].

## Results

The total cost to implement each of the three salt substitution strategies by year of implementation is shown in Table 5. The cost of the subsidised strategy started at 13,678,227,816 ₫ (US$ 589,313) in year 1, increased to 30,539,726,723 ₫ (US$ 1,326,193) in year 2 and decreased to an ongoing cost of 21,808,968,902 ₫ (US$ 977,354) for years 6+. The cost of the regulatory strategy started at 12,186,495,637 ₫ (US$ 525,043) in year 1, increased to 17,311,069,141 ₫ (US$ 751,688) in year 2 and decreased to an ongoing cost of 12,949,953,247 ₫ (US$ 580,410) for years 6 +.

**Table 5:** Total cost of programme implementation phases (per annum) for each salt substitution strategy as estimated through the cost-effectiveness model, Vietnam

Phase	Voluntary	Subsidised	Regulatory
Planning (Year 1)	0 ₫ (US$ 0)	13,678,227,816 ₫ (US$ 589,313)	12,186,495,637 ₫ (US$ 525,043)
Development (Year 2)	0 ₫ (US$ 0)	30,539,726,723 ₫ (US$ 1,326,193	17,311,069,141 ₫ (US$ 751,688)
Partial implementation (Years 3–5)	0 ₫ (US$ 0)	24,336,782,722 ₫ (US$ 1,073,542)	12,843,983,050 ₫ (US$ 566,570)
Full implementation (Years 6+)	0 ₫ (US$ 0)	21,808,968,902 ₫ (US$ 977,354)	12,949,953,247 ₫ (US$ 580,410)

On a yearly basis, the total cost of the voluntary, subsidised and regulatory strategies was estimated to be 0 ₫ (US$ 0.00), 536 ₫ (US$ 0.02) and 37 ₫ (US$ 0.002) per capita, respectively. However, these costs were offset by healthcare savings due to reduced salt intake and reduced stoke and IHD events. Overall the yearly per capita savings across the three salt substitute scenarios was 49 ₫ (voluntary; US$ 0.002), 617 ₫ (subsidised; US$ 0.03) and 3479 ₫ (regulatory; US$ 0.15) (Table 6). Extrapolated to a population level, the yearly cost savings would be between 4775 m ₫ (US$ 205,764) for the voluntary strategy and 337,603 m ₫ (US$ 14,545,300) for the regulatory strategy.

**Table 6:** Estimated costs accrued with each salt substitution strategy (per capita per annum) from the cost-effectiveness model, Vietnam

Parameter	No intervention	Voluntary	Subsidised	Regulatory
Average salt substitute strategy costs per capita per year	0 ₫	0 ₫	63 ₫ (US$ 0.002)	37 ₫ (US$ 0.002)
Average salt reformulation cost per capita per year	0 ₫	0 ₫	473 ₫ (US$ 0.02)	0 ₫
Average healthcare cost per capita per year	15,050 ₫ (US$ 0.65)	15,001 ₫ (US$ 0.65)	13,896 ₫ (US$ 0.62)	11,534 ₫ (US$ 0.50)
**Average total cost per capita per year**	**15,050 ₫ (US$ 0.65)**	**15,001 ₫ (US$ 0.65)**	**14,433 ₫ (US$ 0.62)**	**11,571 ₫ (US$ 0.50)**
***Average total incremental cost*** **per capita** ***per year***		***−49 ₫ (US$ 0.002)***	***−617 ₫ (US$ 0.03)***	***−3479 ₫ (US$ 0.15)***
***Total incremental savings per year***^**a**^		***4775 m ₫ (US$ 205,764)***	***59,873 m ₫ (US$ 2,579,547)***	***337,603 m ₫ (US$ 14,545,300)***

^a^Assuming population of 97,040,334

Relative to no intervention, all three of the salt substitution strategies were found to result in less costs and more QALYs gained over a lifetime. Savings and health gains were driven by reductions in stroke and IHD events. Over the model lifetime (~ 70 years), the voluntary strategy avoided 32,595 and 22,830 stroke and IHD events, respectively. The subsidised strategy avoided 768,384 and 537,157 stroke and IHD events respectively, and finally, the regulatory strategy avoided 2,366,480 and 1,648,590 stroke and IHD events respectively (Table 7).

**Table 7:** Base case cost-effectiveness results of the salt substitution strategies from the cost-effectiveness model, Vietnam

Strategy	Cost	IncrementalCost	Strokes avoided	IHD events avoided	QALYs gained	Incremental Effectiveness	ICER
No intervention	1,053,481 ₫ (US$ 45.39)	–	–	–	13.33	–	–
Voluntary	1,050,036 ₫ (US$ 45.24)	−3445 ₫ (−US$ 0.15)	32,595	22,830	13.34	0.009	DOMINANT
Subsidised	1,010,292 ₫ (US$ 43.53)	−43,189 ₫ (−US$ 1.86)	768,384	537,157	13.35	0.022	DOMINANT
Regulatory	809,951 ₫ (US$ 34.90)	−243,530 ₫ (−US$ 10.49)	236,6480	1,648,590	13.41	0.074	DOMINANT

Abbreviations: *ICER* incremental cost-effectiveness ratio, *IHD* ischaemic heart disease, *QALY* quality adjusted life year

While all three strategies reduced average government costs, the voluntary salt substitution strategy provided the smallest average cost reduction (3445 ₫; US$ 0.15) and effectiveness benefit (0.009 incremental QALYs gained), as it provided the lowest reduction in dietary sodium intake and thus the lowest reduction in IHD or stroke risk. The subsidised strategy provided an average cost-saving of 43,189₫ (US$ 1.86) and resulted in an average incremental QALY gain of 0.022. The regulatory strategy provided the highest cost savings (243,530 ₫; US$ 10.49) and incremental QALYs gained (0.074) (Table 7).

**Fig. 2:**
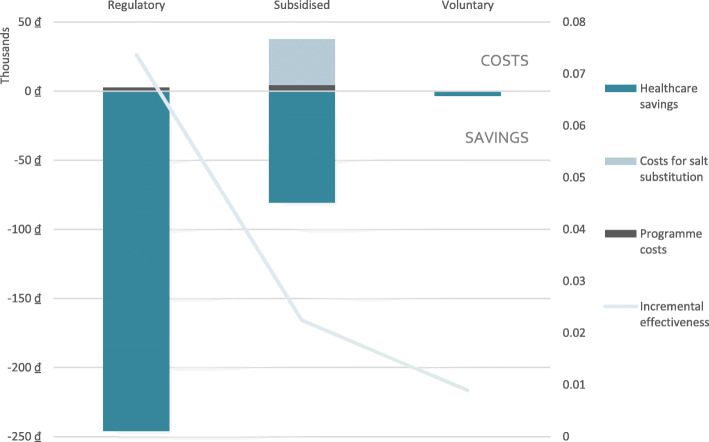
Average per capita incremental cost and effectiveness of each salt substitute strategy as calculated through the cost-effectiveness model, Vietnam

As shown in Fig. 2, savings from reduced healthcare offset implementation costs for all three salt substitution strategies. The savings and effectiveness increase when moving from the voluntary strategy to the subsidised strategy to the regulatory strategy due to the increasing population coverage of the reformulated products.

### Sensitivity and threshold analyses

The results of the sensitivity analysis for the voluntary, subsidised and regulatory strategies compared to no intervention are presented in detail in the Supplementary Material (see Supplementary Tables 5–7). Overall, all strategies proved to be robust to all parameter changes, with all three salt substitution strategies remaining dominant (less costly and more effective) for all scenarios with the exception of removing all Government healthcare costs.

Threshold analysis results are presented in Table 8. For the voluntary strategy the average daily salt reduction required for costs to equal savings was < 0.01 g at all time horizons. Due to the larger government investment in the subsidised strategy, the average salt reduction for costs to equal savings was 2.17 g, 0.27 g and 0.15 g at 20 years, 40 years and over a lifetime respectively (the reduction required at 10 years exceed the average daily intake). For the regulatory strategy, the average salt reduction for costs to equal savings was 1.44 g, 0.18 g, 0.05 g and 0.04 g at 10 years, 20 years, 40 years and over a lifetime respectively.

**Table 8:** Threshold analyses on average daily salt reduction for costs to equal savings for each salt substitution strategy from the cost-effectiveness model, Vietnam

Time horizon	Voluntary	Subsidised	Regulatory
10-years	< 0.01 g	NA	1.44 g
20-years	< 0.01 g	2.17 g	0.18 g
40-years	< 0.01 g	0.27 g	0.05 g
Lifetime	0 g	0.15 g	0.04 g

NA: The salt reduction required for costs to equal savings was greater than the average daily intake

## Discussion

The results of this analysis demonstrate the economic impact of three different population level salt substitution strategies. Overall, all strategies are considered cost-effective and good value for money from a Vietnam Government perspective. This is due to the low cost of implementing salt substitution strategies at a population level and the considerable costs avoided from initial and long term treatment of stroke and IHD events. Specifically, the annual cost of implementing salt substitution strategies was equal to or less than 536 ₫ (US$ 0.02) per captia but led to a reduction of between 32,595 and 2,366,480 strokes and between 22,830 and 1,648,590 IHD events over the lifetime of the model (~ 70 years). The reduction in stroke and IHD events led to substantial healthcare savings, making the salt substitution less costs and more effective than the status quo.

The regulatory strategy proved to have the greatest cost-saving, as the Government did not incur the cost of subsidising potassium chloride as well as media and communication costs. The regulatory strategy also provided the greatest quality of life benefit compared to no intervention. This is due to the regulatory strategy having the greatest coverage and impact on the target population blood pressure, leading to over 2.3 m and 1.6 m stroke and IHD events avoided. Nonetheless, the regulatory strategy would require a concerted policy-making process to implement, including the appropriate planning, implementation, and enforcement legislation. If a less rigorous option is preferred, our analysis also shows the voluntary and subsidised strategies would also provide significant benefits and cost savings to the Vietnam population.

Our results are in line with a recent systematic review of cost-effectiveness publications of different interventions to reduce salt consumption, which found 59 of 62 identified scenarios to be cost-saving [52]. A previous cost-effectiveness analysis of population level interventions aimed at preventing CVD in Vietnam found all programmes to be very cost-effective according to the classification of Commission on Macroeconomics and Health (CMH) on cost-effectiveness [13]. Due to the scarcity of resources available to low and middle income countries such as Vietnam, Ha et al. determined that a health education programme to reduce salt intake and a combined mass media programme on salt, tobacco and cholesterol were the most cost-effective measures [13]. The results of Ha et al. rely on reducing salt consumption through behaviour change whereas our study considers the broader impact of salt substitution policies, demonstrating how various levels of population wide salt reduction interventions are good value for money.

Webb et al. [26] previously studied the cost-effectiveness of implementing a national policy comprising of industry agreement, Government monitoring and public education to decrease sodium intake in 183 nations. The research determined cost-effectiveness of the intervention to be greatest in South Asia, however the study did not take into consideration healthcare costs. For Vietnam, the model estimated 246,143 DALYs averted over 10 years with a cost per capita of US$ 0.31. When including healthcare savings from averted stroke and IHD events, our analysis finds such a programme to be even more cost-effective. For the subsidised programme with a 10-year time horizon excluding healthcare costs and with programme implementation costs only shared by those who potentially receive a health benefit from the intervention (i.e. population aged over 25), the cost per capita is approximately US$ 0.25.

This analysis has several important limitations. Firstly, we do not have data on the true effect of the salt substitution strategies in Vietnam and therefore all scenarios are hypothetical and may not reflect the final characteristics of voluntary, subsidised and regulatory strategies. This limitation is not unique to our research as previous economic evaluations have all included theoretical scenarios and assumptions concerning effectiveness of salt reduction (see Supplementary Material Table 8 for a comparison vs previous literature [13, 18, 26]). To address this, we conducted multiple sensitivity analyses and a threshold analysis to establish a break-even point for each salt substitution strategy. Collectively, these analyses demonstrate that a range of salt substitution strategies are cost-effective and only modest reductions in salt reduction is required at a population level for costs to equal savings.

This analysis was undertaken from the perspective of the Vietnam Government and therefore costs being shifted to industry and consumers are not considered in the analysis. A societal perspective would factor in these initial intervention costs but would to some extent be offset by the savings in out of pocket and private sector costs associated with the prevention of CVD events. The variability in costs of healthcare in Vietnam and the introduction of the universal healthcare scheme also brings uncertainty to the cost of healthcare to the Government. In 2005, the Government on average covered approximately 32% of treatment costs [53], but this has since increased to approximately half and is expected to increase further in the coming years. Taking this into consideration, a sodium reduction programme that reduces stroke and IHD events would be even more cost-effective as the government pays an increasingly higher share of the healthcare costs. The cost of care in Vietnam also varies greatly on factors such as geographical location [54], and as the costing studies used in this model are generally from hospitals in major urban cities the cost may not be representative of the true cost of stroke and IHD treatment in non-urban areas in Vietnam.

This model does not take into consideration additional negative health consequences of potassium reformulated salt, for example the risk of hyperkalaemia in people with advanced kidney disease or diabetes [55]. On balance however, the model does not take into consideration the known beneficial effect of potassium on blood pressure in the general population [56] nor does it consider the lowered risk of stomach cancer [57]. Previous research in China showed salt substitution with potassium chloride would potentially cause 11,000 more deaths due to hyperkalaemia but prevent over 460,000 deaths from cardiovascular disease [4]. Therefore, the omission of the negative impact of potassium chloride is unlikely to bias the results.

## Conclusion

This research shows that a range of modelled salt substitution strategies would be cost-effective for the Vietnam Government in lowering SBP at a population level and consequently lowering the risk of IHD and stroke. The regulatory intervention provides the most cost-effective option, however, it may not fit within the current government approach or could be met with industry and consumer group opposition. The subsidised alternative would require a higher level of government investment, however the implementation costs will be exceeded by healthcare savings assuming a reasonable time horizon is considered. Considering the high cost of healthcare and the low cost of programme implementation, the Vietnamese Government should strongly consider employing a population level intervention to substitute salt with potassium chloride.

## Supplementary Information

**Additional file 1: Supplementary Table 1.** Estimated systolic blood pressure according to salt substitute strategy and age strata, Vietnam. **Supplementary Table 2.** Baseline incidence of stroke and IHD according to salt substitute strategy and age strata, Vietnam. **Supplementary Table 3.** Relative risk reduction of stroke according to salt substitute strategy and age strata, Vietnam. **Supplementary Table 4**. Relative risk reduction of IHD according to salt substitute strategy and age strata, Vietnam. **Supplementary Table 5**. Results of the sensitivity analysis: Regulatory strategy. **Supplementary Table 6**. Results of the sensitivity analysis: Subsidised strategy. **Supplementary Table 7**. Results of the sensitivity analysis: Voluntary strategy. **Supplementary Table 8**. Comparison of cost-effectiveness studies

## Data Availability

The datasets used and/or analysed during the current study are available from the corresponding author on reasonable request.

## Abbreviations[

CER: Cost-effectiveness ratio
CMH: Commission on Macroeconomics and Health
CVD: Cardiovascular disease
DALY: Disability adjusted life year
GBD: Global Burden of Disease study
ICER: Incremental cost-effectiveness ratio
IHD: Ischaemic heart disease
MBS: Medicare Benefits Schedule
NCD: Non communicable disease
PBS: Pharmaceutical Benefits Schedule
PPP: Purchasing power parity
QALY: Quality adjusted life year
RR: Relative risk
SBP: Systolic blood pressure
UN-EU: United Nations Agencies in Vietnam, European Union
VND: Vietnamese Dong
WHO: World Health Organisation
