# Supplementary material for: The cost-effectiveness of government actions to reduce sodium intake through salt substitutes in Vietnam
Source: Arch Public Health. 2021 Mar 11;79:32. doi: 10.1186/s13690-021-00540-4 (PMC7953693; doi:10.1186/s13690-021-00540-4)
Supplement: Supplementary file 1 — Additional file 1: Supplementary Table 1. Estimated systolic blood pressure according to salt substitute strategy and age strata, Vietnam. Supplementary Table 2. Baseline incidence of stroke and IHD according to salt substitute strategy and age strata, Vietnam. Supplementary Table 3. Relative risk reduction of stroke according to salt substitute strategy and age strata, Vietnam. Supplementary Table 4. Relative risk reduction of IHD according to salt substitute strategy and age strata, Vietnam. Supplementary Table 5. Results of the sensitivity analysis: Regulatory strategy. Supplementary Table 6. Results of the sensitivity analysis: Subsidised strategy. Supplementary Table 7. Results of the sensitivity analysis: Voluntary strategy. Supplementary Table 8. Comparison of cost-effectiveness studies [file 13690_2021_540_MOESM1_ESM.docx]

**Supplementary material**

Baseline systolic blood pressure (SBP) was stratified by age and sex, derived from a cost-effectiveness analysis of cardiovascular disease prevention interventions in Vietnam [13]. The age and gender specific SBP for each programme was calculated linearly using the percent sodium reduction from baseline and the estimated mean SBP at zero sodium in economically undeveloped countries [32]. The estimated SBP for each programme is presented in Table 6.

Supplementary Table 1 Estimated systolic blood pressure according to salt substitute strategy and age strata, Vietnam

| Age | Baseline | Voluntary | Subsidised | Regulatory |
| --- | --- | --- | --- | --- |
| 30-44 | 113.77 | 113.75 | 113.22 | 112.15 |
| 45-59 | 124.11 | 124.02 | 122.08 | 118.14 |
| 60-69 | 132.74 | 132.60 | 129.48 | 123.15 |
| 70-79 | 136.15 | 135.99 | 132.40 | 125.13 |
| 80+ | 136.15 | 135.99 | 132.40 | 125.13 |

Note: sex standardised using Vietnam population statistic of 49% male

Source: Ha 2011, WHO-CHOICE

In order to calculate the reduced risk of stroke and IHD due to a reduction in SBP, the baseline incidence of stroke and IHD events in the Vietnamese population was stratified by age and sex as published by Ha 2011, presented in Table 7. A risk reduction was then applied to each baseline incidence, based on the decrease in SBP.

Supplementary Table 2 Baseline incidence of stroke and IHD according to salt substitute strategy and age strata, Vietnam

| Age | Stroke | | IHD | |
| --- | --- | --- | --- | --- |
|  | **Male** | **Female** | **Male** | **Female** |
| 30-44 | 0.00010 | 0.00030 | 0.00003 | 0.00050 |
| 45-59 | 0.00050 | 0.00300 | 0.00100 | 0.00450 |
| 60-69 | 0.00190 | 0.01230 | 0.00210 | 0.02000 |
| 70-79 | 0.00210 | 0.03260 | 0.00230 | 0.04090 |
| 80+ | 0.00350 | 0.05320 | 0.00440 | 0.05760 |

Source: Ha 2011

A network meta-analysis used in previous cost-effectiveness studies [17] estimated a 6.3% reduction in the incidence of stroke and 3.4% reduction in the incidence of IHD for every 1% change in SBP [56]. Accordingly, the relative risk reduction for stroke and IHD for each programme according to the reduction in SBP at each age group, is presented in Table 8 and Table 9, respectively.

Supplementary Table 3 Relative risk reduction of stroke according to salt substitute strategy and age strata, Vietnam

| Age | Voluntary | Subsidised | Regulatory |
| --- | --- | --- | --- |
| 30-44 | 0.99867 | 0.96939 | 0.90995 |
| 45-59 | 0.99554 | 0.89703 | 0.69708 |
| 60-69 | 0.99329 | 0.84524 | 0.54473 |
| 70-79 | 0.99249 | 0.82658 | 0.48983 |
| 80+ | 0.99249 | 0.82658 | 0.48983 |

Source: Calculated from Ha 2011 and Cobiac 2012

The greatest relative risk reduction was observed in the population aged 70 and older under the regulatory programme, whereby the incidence of stroke was reduced by approximately 50 percent. Similarly, for IHD events, incidence was approximately 30 percent lower in the same subgroup.

Supplementary Table 4 Relative risk reduction of IHD according to salt substitute strategy and age strata, Vietnam

| Age | Voluntary | Subsidised | Regulatory |
| --- | --- | --- | --- |
| 30-44 | 0.99928 | 0.98348 | 0.95140 |
| 45-59 | 0.99759 | 0.94443 | 0.83652 |
| 60-69 | 0.99638 | 0.91648 | 0.75430 |
| 70-79 | 0.99594 | 0.90641 | 0.72467 |
| 80+ | 0.99594 | 0.90641 | 0.72467 |

Source: Calculated from Ha 2011 and Cobiac 2012

Sensitivity analyses.

Supplementary Table 5 Results of the sensitivity analysis: Regulatory strategy

| Parameter | Costs (₫) | | Costs (US$) | | QALYs | | ICER | |
| --- | --- | --- | --- | --- | --- | --- | --- | --- |
| Base case | -243,530 ₫ | | -$10.49 | | 0.0737 | | DOMINANT | |
|  | Lower | Upper | Lower | Upper | lower | upper | lower | upper |
| % Products sodium reduced* | -217,840 ₫ | NA | -$217,840 | NA | 0.0666 | NA | DOMINANT | NA |
| Discount rate costs | -621,987 ₫ | -137,452 ₫ | -$621,987 | -$137,452 | 0.0737 | 0.0737 | DOMINANT | DOMINANT |
| Discount rate QALYs | -243,530 ₫ | -243,530 ₫ | -$243,530 | -$243,530 | 0.2042 | 0.0400 | DOMINANT | DOMINANT |
| Incidence of stroke | -233,774 ₫ | -252,856 ₫ | -$233,774 | -$252,856 | 0.0688 | 0.0771 | DOMINANT | DOMINANT |
| Stroke RR per 1% SBP Δ | -198,839 ₫ | -277,185 ₫ | -$198,839 | -$277,185 | 0.0618 | 0.0827 | DOMINANT | DOMINANT |
| Incidence of IHD | -234,635 ₫ | -252,132 ₫ | -$234,635 | -$252,132 | 0.0723 | 0.0751 | DOMINANT | DOMINANT |
| IHD RR per 1% SBP Δ | -150,014 ₫ | -307,442 ₫ | -$150,014 | -$307,442 | 0.0470 | 0.0918 | DOMINANT | DOMINANT |
| Event cost Govt: Stroke | -118,565 ₫ | -339,845 ₫ | -$118,565 | -$339,845 | 0.0737 | 0.0737 | DOMINANT | DOMINANT |
| Event cost Govt: IHD | -125,049 ₫ | -344,053 ₫ | -$125,049 | -$344,053 | 0.0737 | 0.0737 | DOMINANT | DOMINANT |
| Long term Govt cost: Stroke | -243,530 ₫ | -967,996 ₫ | -$243,530 | -$967,996 | 0.0737 | 0.0737 | DOMINANT | DOMINANT |
| Long term Govt cost: IHD | -240,846 ₫ | -245,808 ₫ | -$240,846 | -$245,808 | 0.0737 | 0.0737 | DOMINANT | DOMINANT |
| Cost of salt substitution/KG | -243,530 ₫ | -243,530 ₫ | -$243,530 | -$243,530 | 0.0737 | 0.0737 | DOMINANT | DOMINANT |
| Cost of healthcare to Govt | 2,601 ₫ | -452,355 ₫ | $2,601 | -$452,355 | 0.0737 | 0.0737 | 35,291 ₫ | DOMINANT |
| Cost project implementation | -244,050 ₫ | -243,010 ₫ | -$244,050 | -$243,010 | 0.0737 | 0.0737 | DOMINANT | DOMINANT |
| Disutility stroke event | -243,530 ₫ | -243,530 ₫ | -$243,530 | -$243,530 | 0.0731 | 0.0745 | DOMINANT | DOMINANT |
| Disutility IHD event | -243,530 ₫ | -243,530 ₫ | -$243,530 | -$243,530 | 0.0731 | 0.0744 | DOMINANT | DOMINANT |
| Utility long-term stroke | -243,530 ₫ | -243,530 ₫ | -$243,530 | -$243,530 | 0.0756 | 0.0718 | DOMINANT | DOMINANT |
| Utility long term IHD | -243,530 ₫ | -243,530 ₫ | -$243,530 | -$243,530 | 0.0737 | 0.0737 | DOMINANT | DOMINANT |
| Mortality of stroke | -243,529 ₫ | -243,531 ₫ | -$243,529 | -$243,531 | 0.0726 | 0.0748 | DOMINANT | DOMINANT |
| Mortality of IHD | -244,044 ₫ | -243,016 ₫ | -$244,044 | -$243,016 | 0.0717 | 0.0758 | DOMINANT | DOMINANT |

Abbreviations: IHD, ischaemic heart disease; QALYs, quality adjusted life years; SBP, systolic blood pressure

*An upper scenario was not included as the value would exceed 100%

Supplementary Table 6 Results of the sensitivity analysis: Subsidised strategy

| Parameter | Costs (₫) | | Costs (US$) | | QALYs | | ICER | |
| --- | --- | --- | --- | --- | --- | --- | --- | --- |
| Base case | -43,189 ₫ | | -$1.86 | | 0.0225 | | DOMINANT | |
|  | lower | upper | lower | upper | lower | upper | lower | upper |
| % Products sodium reduced | -39,261 ₫ | -47,159 ₫ | -$39,261 | -$47,159 | 0.0195 | 0.0254 | DOMINANT | DOMINANT |
| Discount rate costs | -144,736 ₫ | -16,448 ₫ | -$144,736 | -$16,448 | 0.0225 | 0.0225 | DOMINANT | DOMINANT |
| Discount rate QALYs | -43,189 ₫ | -43,189 ₫ | -$43,189 | -$43,189 | 0.0645 | 0.0117 | DOMINANT | DOMINANT |
| Incidence of stroke | -40,070 ₫ | -46,140 ₫ | -$40,070 | -$46,140 | 0.0215 | 0.0233 | DOMINANT | DOMINANT |
| Stroke RR per 1% SBP Δ | -29,000 ₫ | -53,741 ₫ | -$29,000 | -$53,741 | 0.0185 | 0.0254 | DOMINANT | DOMINANT |
| Incidence of IHD | -40,327 ₫ | -45,947 ₫ | -$40,327 | -$45,947 | 0.0215 | 0.0234 | DOMINANT | DOMINANT |
| IHD RR per 1% SBP Δ | -12,979 ₫ | -63,493 ₫ | -$12,979 | -$63,493 | 0.0141 | 0.0280 | DOMINANT | DOMINANT |
| Event cost Govt: Stroke | -2,251 ₫ | -74,742 ₫ | -$2,251 | -$74,742 | 0.0225 | 0.0225 | DOMINANT | DOMINANT |
| Event cost Govt: IHD | -4,262 ₫ | -76,216 ₫ | -$4,262 | -$76,216 | 0.0225 | 0.0225 | DOMINANT | DOMINANT |
| Long term Govt cost: Stroke | -43,189 ₫ | -468,908 ₫ | -$43,189 | -$468,908 | 0.0225 | 0.0225 | DOMINANT | DOMINANT |
| Long term Govt cost: IHD | -42,307 ₫ | -43,937 ₫ | -$42,307 | -$43,937 | 0.0225 | 0.0225 | DOMINANT | DOMINANT |
| Cost of salt substitution/KG | -59,758 ₫ | -10,052 ₫ | -$59,758 | -$10,052 | 0.0225 | 0.0225 | DOMINANT | DOMINANT |
| Cost of healthcare to Govt | 37,559 ₫ | -111,698 ₫ | 37,559 | -$111,698 | 0.0225 | 0.0225 | 1,672,529 ₫ | DOMINANT |
| Cost project implementation | -44,073 ₫ | -42,305 ₫ | -$44,073 | -$42,305 | 0.0225 | 0.0225 | DOMINANT | DOMINANT |
| Disutility stroke event | -43,189 ₫ | -43,189 ₫ | -$43,189 | -$43,189 | 0.0223 | 0.0227 | DOMINANT | DOMINANT |
| Disutility IHD event | -43,189 ₫ | -43,189 ₫ | -$43,189 | -$43,189 | 0.0223 | 0.0227 | DOMINANT | DOMINANT |
| Utility long-term stroke | -43,189 ₫ | -43,189 ₫ | -$43,189 | -$43,189 | 0.0231 | 0.0218 | DOMINANT | DOMINANT |
| Utility long term IHD | -43,189 ₫ | -43,189 ₫ | -$43,189 | -$43,189 | 0.0225 | 0.0225 | DOMINANT | DOMINANT |
| Mortality of stroke | -43,179 ₫ | -43,199 ₫ | -$43,179 | -$43,199 | 0.0221 | 0.0228 | DOMINANT | DOMINANT |
| Mortality of IHD | -43,343 ₫ | -43,035 ₫ | -$43,343 | -$43,035 | 0.0221 | 0.0228 | DOMINANT | DOMINANT |

Abbreviations: IHD, ischaemic heart disease; QALYs, quality adjusted life years; SBP, systolic blood pressure

Supplementary Table 7 Results of the sensitivity analysis: Voluntary strategy

| Parameter | Costs (₫) | | Costs (US$) | | QALYs | | ICER | |
| --- | --- | --- | --- | --- | --- | --- | --- | --- |
| Base case | -3,445 ₫ | | -$0.49 | | 0.0089 | | DOMINANT | |
|  | lower | upper | lower | upper | lower | upper | lower | upper |
| % Products sodium reduced | -2,296 ₫ | -4,595 ₫ | -$2,296 | -$4,595 | 0.0086 | 0.0092 | DOMINANT | DOMINANT |
| Discount rate costs | -8,646 ₫ | -1,966 ₫ | -$8,646 | -$1,966 | 0.0089 | 0.0089 | DOMINANT | DOMINANT |
| Discount rate QALYs | -3,445 ₫ | -3,445 ₫ | -$3,445 | -$3,445 | 0.0156 | 0.0066 | DOMINANT | DOMINANT |
| Incidence of stroke | -3,316 ₫ | -3,567 ₫ | -$3,316 | -$3,567 | 0.0089 | 0.0089 | DOMINANT | DOMINANT |
| Stroke RR per 1% SBP Δ | -2,849 ₫ | -3,886 ₫ | -$2,849 | -$3,886 | 0.0088 | 0.0090 | DOMINANT | DOMINANT |
| Incidence of IHD | -3,327 ₫ | -3,559 ₫ | -$3,327 | -$3,559 | 0.0089 | 0.0089 | DOMINANT | DOMINANT |
| IHD RR per 1% SBP Δ | -2,165 ₫ | -4,299 ₫ | -$2,165 | -$4,299 | 0.0086 | 0.0092 | DOMINANT | DOMINANT |
| Event cost Govt: Stroke | -1,700 ₫ | -4,790 ₫ | -$1,700 | -$4,790 | 0.0089 | 0.0089 | DOMINANT | DOMINANT |
| Event cost Govt: IHD | -1,783 ₫ | -4,855 ₫ | -$1,783 | -$4,855 | 0.0089 | 0.0089 | DOMINANT | DOMINANT |
| Long term Govt cost: Stroke | -3,445 ₫ | -13,764 ₫ | -$3,445 | -$13,764 | 0.0089 | 0.0089 | DOMINANT | DOMINANT |
| Long term Govt cost: IHD | -3,407 ₫ | -3,477 ₫ | -$3,407 | -$3,477 | 0.0089 | 0.0089 | DOMINANT | DOMINANT |
| Cost of salt substitution/KG | -3,445 ₫ | -3,445 ₫ | -$3,445 | -$3,445 | 0.0089 | 0.0089 | DOMINANT | DOMINANT |
| Cost of healthcare to Govt | 0 ₫ | -6,368 ₫ | $0 | -$6,368 | 0.0089 | 0.0089 | DOMINANT | DOMINANT |
| Cost project implementation | -3,445 ₫ | -3,445 ₫ | -$3,445 | -$3,445 | 0.0089 | 0.0089 | DOMINANT | DOMINANT |
| Disutility stroke event | -3,445 ₫ | -3,445 ₫ | -$3,445 | -$3,445 | 0.0089112 | 0.0089 | DOMINANT | DOMINANT |
| Disutility IHD event | -3,445 ₫ | -3,445 ₫ | -$3,445 | -$3,445 | 0.0089111 | 0.0089 | DOMINANT | DOMINANT |
| Utility long-term stroke | -3,445 ₫ | -3,445 ₫ | -$3,445 | -$3,445 | 0.0089464 | 0.0088 | DOMINANT | DOMINANT |
| Utility long term IHD | -3,445 ₫ | -3,445 ₫ | -$3,445 | -$3,445 | 0.0089193 | 0.0089 | DOMINANT | DOMINANT |
| Mortality of stroke | -3,445 ₫ | -3,445 ₫ | -$3,445 | -$3,445 | 0.0089 | 0.0089 | DOMINANT | DOMINANT |
| Mortality of IHD | -3,452 ₫ | -3,438 ₫ | -$3,452 | -$3,438 | 0.0089 | 0.0089 | DOMINANT | DOMINANT |

Abbreviations: IHD, ischaemic heart disease; QALYs, quality adjusted life years; SBP, systolic blood pressure

Validation

Supplementary Table 8 is provided to enable a comparison of similar cost-effectiveness studies examining salt reduction or regulation

Supplementary Table 8 Comparison of cost-effectiveness studies

|  | Ha 2011 | Webb 2017 | Cobiac 2010 | Current analysis |
| --- | --- | --- | --- | --- |
| Intervention | Health education through mass media to reduce salt intake and a voluntary reduction in salt content of processed foods.  Education and individual treatment (b-blocker and diuretic) for high systolic blood pressure (>140mmHg and >160mmHg)  Combination treatment with b-blocker, diuretic, statins and aspirin for individuals with absolute risk of cardiovascular event | Soft regulation policy. Government supported industry agreements to reduce sodium in processed foods, Government monitoring of industry compliance, and a public health campaign targeting consumer choices, including sustained pressure on food manufacturers to pursue progressive reformulation, reinforced by food group specific targets, independent monitoring, and a sustained media campaign against excess salt intake. | Population level community heart health program and mandatory reduction of salt in the manufacture of breads, margarines and cereals. | **Voluntary** introduction of new cooking salt, fish sauce and bot canh with a potassium-fortified reformulation  Provision of **Government subsidy** to manufacturers for replacing high-sodium cooking salt, fish sauce and bot canh with potassium-fortified salt products together with media and communication support  Regulatory approach: Legislation requiring **compulsory** replacement of salt with potassium chloride, learning from universal iodisation program experience |
| Time Horizon | 10 Year | 10 Year closed cohort | Lifetime (35-84) | Lifetime (ages 30-100) |
| Model structure | Health gains: WHO-CHOICE PopMod  Costs: WHO-CHOICE CostIT | Health gains: WHO-CHOICE PopMod  Costs: WHO-CHOICE CostIT | Markov model | |
| Implementation phasing: Effectiveness | Not specified (assumed instant effect) | Effectiveness: “soft regulation” policy: Linear 10% reduction in Na over 10 years. No lag in benefits from SBP reduction and CVD | Not specified (assumed instant effect) | Modified Webb 2017  Years 1-2  All programmes: Planning and development stage, no impact of intervention on SBP  Years 3-5  Voluntary and subsidised: Partial implementation stage, 50% impact compared to full implementation  Regulatory: Full impact of intervention on SBP  Years 6+:  All programmes: Full impact of intervention on SBP |
| Implementation phasing: Costs | Not specified | Year 1: Planning  Year 2: Development  Years 3-5: Partial implementation  Years 6-10: Full implementation | No phasing for mandatory or voluntary salt reduction | Modified Webb 2017  Years 1-2:  Planning and development  Years 3-5:  Voluntary and subsidised: Partial implementation  Regulatory: Full implementation  Years 6+:  Full implementation |
| Currency | ₫ | (I$) International dollars | AU$ | ₫ and US$ |
| Discounting | 3% | 3% | 3% | 3% |
| Perspective | Individual and national level intervention: includes societal costs | National Government: program costs only | National Government: program and healthcare costs | National Government: programme and healthcare costs (54% of healthcare costs are borne by the Government) |
| Source of resource use | Not referenced. Likely input from local experts | WHO-CHOICE | WHO-CHOICE | As Webb 2017 |
| Source of resource costs | UN-EU Vietnam and other local sources | WHO-CHOICE | Voluntary salt reduction: Heart Foundation annual fee per product (data not published). Legislative and enforcement: WHO unit costs | UN-EU Vietnam local cost norms 2015  (as Ha 2011 but updated figures)  Salt production: 534,798 tonnes (Vietnam The Ministry of Agriculture and Rural Development)  Cost of salt reformulation: US$0.04/kg (iodisation used as proxy) |
| Source of healthcare costs | Drugs: International Drug Price database  Diagnostic tests: Not specified  Primary and secondary care: Flessa and Dung 2004 (Vietnam specific)  Salaries: UN-EU 2007 | Healthcare costs not included | MBS, PBS | Initial stroke event: Khiaocharoen 2012 (Thailand)  Long-term cost of stroke: No long term cost (Nguyen 2016)  Initial IHD event: Nguyen 2016 (Vietnam)  Long-term cost of stroke: Nguyen 2016 (Vietnam) |
| Event risk reduction | WHO Comparative Risk Assessment Project (Ezzati 2004) | Hypertensive status calculated from the effect of Na reduction on SBP, stratified by age and sex. Effect of SBP reduction on CVD by age | Change in disease incidence with 1% change in SBP: IHD = 3.4%, Stroke 6.3% | As Cobiac 2012. Ha 2011 SBP used as baseline. |
| Overall mortality | United Nations Population Division Bureau 2007 | Global Burden of Disease study 2010 (GBD 2010) | Australian Bureau of Statistics minus IHD and stroke related deaths recorded in the Australian Burden of Disease study | WHO Vietnam life tables 2017 |
| Event specific mortality | Not explicitly specified, likely United Nations Population Division Bureau 2007 | WHO-CHOICE | Western Australia Linked Hospital Database. Age and gender specific. 28 day morality and post-28 day mortality | Stroke event: Tirschwell 2012, Vietnam specific  Stroke long term: Kiyohara 2003, Japan relative risk compared to healthy controls  IHD event: WHO-CHOICE  IHD long term: Tang 2007, New Zealand |
| Source of quality of life values | National burden of disease study Vietnam (data not published) | GBD 2010 | Beaver dam study (US population). Age and sex specific. IHD first 6 weeks, IHD post 6 weeks and stroke. | Healthy: Nguyen 2015, Vietnam, age and sex specific  Stage 1 blood pressure: Nguyen 2015, Vietnam, age and sex specific  Stroke event (disutility): GBD 2010  Post stroke: Luengo-Fernandez 2013  IHD event (disutility): GBD 2010  Post IHD: Nguyen 2014 Vietnam specific history of event odds ratio |
| Results | Media salt campaign  Costs per year: 89 billion ₫  DALYs averted: 45,939  CER: 1,945,002₫ (US$118) per DALY averted (CMH category: very cost-effective) | Soft regulation policy  Cost per capita: I$0.31  DALYs averted: 246,143  CER: I$62.00 | Mandatory salt reduction  Cost per capita: AU$0.81  DALYs averted: 80,000  ICER: Dominant | **No intervention**  Cost p.c.: 1,053,481 ₫ (US$45.39)  QALY: 13.33  **Regulatory**  Cost p.c.: 809,951 ₫ (US$34.49)  QALY: 13.41  ICER: Dominant  **Subsidised**  Cost p.c.: 1,010,292 ₫ (US$43.53)  QALY: 13.35  ICER: Dominant  **Voluntary**  Cost p.c.: 1,050,036 ₫ (US$45.24)  QALY: 13.34  ICER: Dominant |

Abbreviations: CER, cost-effectiveness ratio; CVD, cardiovascular disease; DALY, disability adjusted life-year; GBD, global burden of disease study; ICER, incremental cost-effectiveness ratio; IHD, ischaemic heart disease; MBS: Medicare Benefits Scheme; PBS, Pharmaceutical Benefits Scheme; p.c., per capita; SBP, systolic blood pressure; WHO, World Health Organisation

Source: Ha and Chisholm [13]; Webb, Fahimi [26]; Cobiac, Magnus [17]
